# Exercise in Children during Health and Sickness

**DOI:** 10.1155/2010/842537

**Published:** 2010-12-29

**Authors:** Mutasim Abu-Hasan, Neil Armstrong, Lars B. Andersen, Miles Weinberger, Patricia A. Nixon

**Affiliations:** ^1^Pediatric, Pulmonary, and Allergy Division, University of Florida, Gainesville, FL 32610-0296, USA; ^2^Children's Health and Exercise Research Centre, School of Sport and Health Sciences, University of Exeter, Exeter EX4 4QJ, UK; ^3^Department Exercise Epidemiology, Institute of Sport Sciences and Clinical Biomechanics, University of Southern Denmark, DK-5270 Odense, Denmark; ^4^Pediatrics, Allergy and Pulmonary Division, Department of Pediatrics, University of Iowa Children's Hospital, Iowa City, IA 52242, USA; ^5^Department of Pediatrics, School of Medicine, Wake Forest University, Winston-Salem, NC 27157, USA

Because of the rapidly increasing prevalence of obesity among children worldwide and the realization that this global epidemic is closely related to changing life style, especially relating to diet and exercise, research in the effects of exercise on children's health and the effects of children's health on their ability to exercise becomes timely and imperative. Promoting scientific researching in the field of exercise medicine in children has therefore been the primary motive behind this special issue of the International Journal of Pediatrics which is dedicated to publishing important works in the field. 

To the great satisfaction of our team of editors, a great number of good quality research results were submitted to the issue from all four corners of the world which indicates a strong interest in this very important field of medicine worldwide. 

The first section of this issue contains cross-sectional population studies that provide evidence confirming the strong correlation between obesity and lack of activity in children of different populations and different age groups (the first, second, and third papers). Furthermore, a 12-month interventional study from Sweden showed that longer exercise periods performed by school age boys resulted in more muscle mass and increased muscle strength (the fourth paper). 

The second section of the issue contains several articles studying the different genetic, environmental, parental, and other psychosocial factors that can potentially affect children's level of exercise activity. These articles collectively provide evidence for the variety and complexity of factors that affect children's predilection or exercise (the fifth, sixth, seventh, and eighth papers). They also identify potential areas for future intervention to promote exercise early in life (the ninth paper). One example of such opportunities involves the use of video gaming. Even though the overuse of video gaming has been largely blamed for the decreasing level of physical activity in children, the case might be completely reversed with the recent advent of interactive video gaming which tends to be preferred by children over conventional video gaming and is associated with higher level of physical activity (the tenth paper). 

The third section deals with research relating to exercise in children with known chronic illness such as diabetes, cystic fibrosis, neuromuscular diseases, arthritis, and congenital heart diseases with emphasis not only concerning the limitations these illnesses impose on children's ability to exercise and become physically fit but also on how increased fitness in these patients can modulate their disease process and therefore on ways exercise can be performed and promoted (the eleventh, twelfth, thirteenth, fourteenth, fifteenth, and sixteenth papers).

The fourth section discusses hemodynamic responses to exercise in children as compared to adults (the seventeenth paper) and explores the hormonal and inflammatory profile of overweight and normal weight children and relates them to cardiovascular fitness (the eighteenth paper). 

The fifth and final section has one article which evaluates the validity of different accelerometeric measurements used in exercise research to objectively grade level of physical activity as compared to the gold standard of directly measuring energy expenditure (the nineteenth paper).

We hope that this special issue will contribute substantially to the existing body of knowledge of this new and growing field of exercise medicine in children and to stimulate further needed research.

*Mutasim Abu-Hasan* *Mutasim Abu-Hasan* *Neil Armstrong* *Neil Armstrong* *Lars B. Andersen* *Lars B. Andersen* *Miles Weinberger* *Miles Weinberger* *Patricia A. Nixon* *Patricia A. Nixon*

